# Informatics solutions for bridging the gap between clinical and laboratory services in a low-resource setting

**DOI:** 10.4102/ajlm.v4i1.176

**Published:** 2015-06-11

**Authors:** Julia Driessen, Henry Limula, Oliver J. Gadabu, Gervase Gamadzi, Edwin Chitandale, Anne Ben-Smith, Noor Alide, Gerald P. Douglas

**Affiliations:** 1 Department of Health Policy and Management, University of Pittsburgh, Pittsburgh, United States; 2 Kamuzu Central Hospital, Ministry of Health, Lilongwe, Malawi; 3 Baobab Health Trust, Lilongwe, Malawi; 4 Department of Biomedical Informatics, University of Pittsburgh, United States; 5 Center for Health Informatics for the Underserved, University of Pittsburgh, United States

## Abstract

**Background:**

There has been little formal analysis of laboratory systems in resource-limited settings, despite widespread consensus around the importance of a strong laboratory infrastructure.

**Objectives:**

This study details the informational challenges faced by the laboratory at Kamuzu Central Hospital, a tertiary health facility in Malawi; and proposes ways in which informatics can bolster the efficiency and role of low-resource laboratory systems.

**Methods:**

We evaluated previously-collected data on three different aspects of laboratory use. A four-week quality audit of laboratory test orders quantified challenges associated with collecting viable specimens for testing. Data on tests run by the laboratory over a one-year period described the magnitude of the demand for laboratory services. Descriptive information about the laboratory workflow identified informational process breakdowns in the pre-analytical and post-analytical phases and was paired with a 24-hour sample of laboratory data on results reporting.

**Results:**

The laboratory conducted 242 242 tests over a 12-month period. The four-week quality audit identified 54% of samples as untestable. Prohibitive paperwork errors were identified in 16% of samples. Laboratory service workflows indicated a potential process breakdown in sample transport and results reporting resulting from the lack of assignment of these tasks to any specific employee cadre. The study of result reporting time showed a mean of almost six hours, with significant variation.

**Conclusions:**

This analysis identified challenges in each phase of laboratory testing. Informatics could improve the management of this information by streamlining test ordering and the communication of test orders to the laboratory and results back to the ordering physician.

## Introduction

### Problem statement

The healthcare challenges faced by resource-limited countries require an efficient and accessible laboratory infrastructure. Laboratory testing plays an irreplaceable role in the diagnosis and treatment of diseases such as HIV, tuberculosis, and malaria.^1^ Monitoring the progression of HIV through laboratory measures such as viral load and CD4 counts is important for managing patients on antiretroviral therapy (ART) and identifying treatment failure. The serious public health threat of drug-resistant tuberculosis also requires laboratory testing for drug sensitivity.^2^ Whilst rapid diagnostic tests are available for malaria and are in use in many health facilities, many countries still consider microscopy performed in the laboratory as the gold standard because it detects a wider array of species.^3^ If laboratory tests are not available or used, there is a substantial risk of inappropriate treatment, which harms the patient, wastes already limited resources and contributes to increased drug resistance.^4,5^

Despite the need for laboratory testing in addressing the infectious disease burden, many laboratories are ill-equipped to play a central role in the diagnostic and care delivery process in resource-constrained countries.^6^ As a result of poor laboratory services and staffing, there is a diagnostic culture amongst physicians of circumventing laboratory testing and using other, less reliable signals to diagnose diseases such as malaria, or treating for malaria despite a negative laboratory result.^7,8,9,10^ In many low-income countries, there is reluctance to order tests such as a sputum microscopy for tuberculosis, since the patient often dies before the results are received.^11^ In Malawi, for example, 40% of hospitals have only one trained laboratory technician.^12,13^ Malawi's national ART programme has relied primarily on clinical criteria for ART treatment initiation.^14^

Whilst compelling, these anecdotal insights and macro-level measures offer an incomplete picture of laboratory testing in low-resource settings and do not indicate how these shortages impact healthcare delivery and health outcomes. For example, investing in additional microscopes may have a limited effect if the necessary reagents are unavailable or physicians rarely order tests that require microscopes. A better understanding of the micro-level dynamics of laboratory testing, as well as the role of the laboratory within a low-resource health system, can identify high-value steps to laboratory strengthening.

#### Key focus

Laboratory testing is typically described as a three-stage process: pre-analytical, analytical and post-analytical.^15,16^ Most studies of laboratory testing, both in low- and high-resource settings, focus on the analytical phase, or the analysis step within the laboratory; it has also been noted that the pre- and post-analytical phases are common sources of delays and errors.^17^ Understanding the dynamics of a low-resource laboratory will also highlight the potential role of informatics in bolstering the efficiency of the laboratory system. Laboratory testing is both materially and informationally intensive. Whereas most coverage of laboratory systems in low-resource settings focuses on the material needs, such as sufficient quantities of reagents and working centrifuges, the information burden is just as demanding, but far less well-understood. Informatics streamlines the management and transfer of data and thus may be appropriate for addressing these information barriers. However, better evidence is needed around the informational demands and barriers experienced in laboratories in low-resource settings.

One problem where informatics solutions have been employed in low-resource settings is in improving clinic access to centralised laboratory testing results.^18,19^ Other systems have been implemented in facilities with on-site laboratories in order to improve management of laboratory histories. In most cases, these systems involve data entry of paper forms, with the goal of maintaining accurate patient histories.^19^

#### Contribution to field

In this article we present detailed data on the role and workflow of clinical laboratory testing at Kamuzu Central Hospital (KCH), an 800+ bed tertiary health facility in Lilongwe, Malawi's capital city, with the goal of understanding other ways that informatics can be used to address laboratory challenges. KCH has been an incubator for developing informatics interventions in the clinical setting since 2001. The hospital has an extensive local area network connecting more than 60 computers across the hospital campus. This is, in turn, connected to a dedicated power backup system and linked to a wireless metropolitan area network spanning greater Lilongwe. Aspects of this work have been described elsewhere.^20,21,22^ Laboratory Systems Strengthening and the promotion of Good Laboratory Practices are supported by a number of technical partners and the laboratory is currently in the process of preparing for an audit under the framework of Strengthening Laboratory Management Toward Accreditation (SLMTA). Four different aspects of the laboratory are assessed: (1) the demand for laboratory testing in the hospital; (2) the burden of untestable samples; (3) the process of laboratory testing in a typical patient visit; and (4) the time required for reporting of results.

## Research method and design

### Setting

KCH is a tertiary care facility in the capital city of Malawi. It serves an area of over four million people, with approximately 50 000 admissions and 245 000 outpatient visits per year.^23^ The laboratory at KCH houses nine different departments: haematology, parasitology, microbiology, molecular biology, serology, flow cytometry, biochemistry, histology and blood bank. The laboratory operates continuously with 27 professional staff, although staffing levels are reduced at nights and on weekends.

### Procedure

We analysed previously-collected data from laboratory records and the results of studies done as part of a quality improvement effort by the KCH laboratory. In 2009, a quality audit was performed on the samples sent for testing to the KCH laboratory over a four-week period.^24^ A total of 3549 samples were evaluated for completeness of the test orders and viability of the samples. If a test order or sample was deemed untestable, the reason for this classification was noted. Issues with test orders included incorrect or incomplete forms and unlabeled samples. Samples were classified as non-viable if the sample quantity was insufficient, or if the samples were clotted, haemolysed, too old, or in the wrong container. Results were stratified by department.

An additional step as part of this quality improvement effort was to analyse the time required for results reporting. This consisted of tracking the amount of time that results were waiting in the laboratory for pick-up over a 24-hour period. A total of 25 patients had test orders sent to the laboratory during this observation period. No patient details were captured for these samples.

Information about test volume was obtained from the KCH laboratory for the time period July 01, 2010 to June 30, 2011. This information is routinely collected by the laboratory and included, for each assay conducted, the total number of tests performed per month. Overall test volume indicates a conservative estimate of the demand for testing in this setting, since some tests could not be performed because of equipment malfunctions and/or reagent shortages. The demand for certain assays is evidence of the types of pressures faced by the laboratory, since different assays have different time sensitivities and testing demands.

We describe the critical steps of the laboratory testing workflow that involve information transfer. The three phases of testing are commonly broken down into nine discrete steps (order, collection, identification, transportation, preparation, analysis, reporting, interpretation, action); and we define the stages within KCH, specifying for each step the location and staff members involved, as well as other pertinent details.^15^ The goal of this exercise is to identify the informational demands and potential process breakdowns in clinical laboratory testing at KCH to better understand the potential role of informatics.

### Ethical considerations

This article describes a rationale for introducing informatics interventions to improve the quality of laboratory services in low-resource settings. The motivation is supported by results from previously-conducted quality improvement audits. No primary research was conducted, thus the work described here does not meet the criteria for requiring institutional review board approval.

## Results

Of the 3549 samples evaluated as part of the quality audit, 54% (*n* = 1923) were not testable (Table 1). There was variation in this rate across the departments within the laboratory, ranging from 5% of samples for microbiology to 70% of samples sent to the blood bank department. An insufficient sample volume was the most common reason for a sample being deemed untestable (*n* = 1606). This characterised over 80% of untestable samples (*n* = 1923) and 45% of all audited samples (*n* = 3549). Of the high number of samples that were considered untestable because of insufficient blood volume, the vast majority came from the paediatric department, where it is challenging to get sufficient blood from a sick and frequently dehydrated infant. Test order forms were filled out either incorrectly or incompletely in just over 16% (*n* = 591) of audited samples. The least frequent problems identified were samples that were mixed up, too old, or in the wrong container.

**TABLE 1:** Results of 2009 quality audit.

Department	Total number of samples	Viable samples (%)	Compromised or discarded samples (%)	Number of compromised / discarded samples that were/had†							
				Haemolysed	Clotted	Insufficient Volume	Old	In incorrect container	Mix-up	Unlabeled	Attached to a form filled out incorrectly/ incompletely
Blood bank	980	290 (30)	690 (70)	73	0	583	4	3	6	21	351
Haematology	1639	568 (35)	1071 (65)	66	29	959	1	1	0	15	168
Microbiology	82	78 (95)	4 (5)	0	0	4	0	0	0	0	12
Biochemistry	848	690 (81)	158 (19)	48	28	60	2	8	0	12	60
**Total**	**3549**	**1626 (46)**	**1923 (54)**	**187**	**57**	**1606**	**7**	**12**	**6**	**48**	**591**

Note: Samples sent to the KCH laboratory were classified by department and viability status. If found to be non-viable, the reason(s) for discarding the sample were documented.

†, Categories are not mutually exclusive.

Between July 01, 2010 and June 30, 2011 the KCH laboratory conducted 242 242 tests (Table 2). The number of tests carried out for the parasitology and blood bank departments accounted for over half of the laboratory's total test workload during this time. The most common tests were: malaria parasites; full blood count; blood grouping and cross matches; and CD4 count. There was considerable monthly variation in the number of tests conducted, reflecting the seasonality of diseases such as malaria. The average monthly test load was 20 187 tests, with a standard deviation of 3263 tests. There were several classes of tests, including blood lipids and hormones, which were not conducted at all during this time period because of inoperative equipment and/or lack of reagents.

**TABLE 2:** Kamuzu Central Hospital laboratory test workload, July 01, 2010 – June 30, 2011.

Department	Total number of tests	Mean number of tests per month	Standard deviation of tests per month	Most ordered test	Frequency of most ordered test
Blood bank	60 206	5017	468	Blood grouping	18 803
Haematology	37 688	3141	499	Full blood count	32 909
Parasitology	70 519	5877	1407	Malaria	66 601
Microbiology	14 743	1229	181	Gram stain	1633
Serology	5613	468	165	Syphilis RPR	3769
Molecular biology	5425	452	450	EID DNA	5123
Flow cytometry	14 090	1174	236	CD4 epic	13 947
Biochemistry	33 199	2767	1247	Blood urea nitrogen	3383
Histology	759	63	11	-	-
**Total**	**242 242**	**20 187**	**3263**	-	-

Note: For each laboratory department, various aspects of the demand for laboratory tests are presented, including the monthly mean of tests conducted, the standard deviation to indicate variability throughout the year, and the most frequent test conducted.

RPR, rapid plasma reagin; EID DNA, early infant diagnosis deoxyribonucleic acid.

**FIGURE 1:**
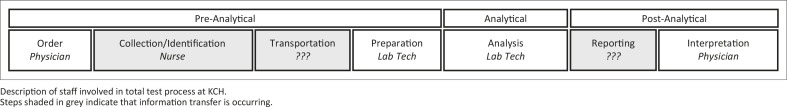
Total testing process workflow at KCH.^1^ Description of staff involved in total test process at KCH. Steps shaded in grey indicate that information transfer is occurring.

Figure 1 defines the total testing process workflow at KCH according to the commonly-specified nine stages of laboratory testing, and indicates the staff responsible for the task.^15,25^ The process begins and ends on the patient ward with the physician, who makes the initial request for a laboratory test and also determines a course of action based on the interpretation of the test results. The pre-analytical phase is initiated with the ordering of a test and also includes the sample collection and identification or matching of the sample with the patient. These two tasks are both completed by a clinician or nurse and are associated with some of the issues identified in the quality audit, such as incomplete or incorrect labeling and sample mix-ups. Incomplete or illegible labeling result from a number of factors, including insufficient label space and lack of necessary information at time of ordering. The pre-analytical phase then extends beyond the patient ward to include transport of the sample to the laboratory and preparation of the sample for testing by a laboratory technician. The transportation phase represents the transfer of the specimen and associated information from one department to another within the hospital; this task is not assigned to a specific job title in the hospital. It is most likely to be carried out by a nurse, a patient attendant, or a janitor, but there is no established routine for sample transport. Samples waiting for transport to the laboratory are typically stored in a treatment room or at a nursing station, and are taken with varying frequency to the laboratory. It is thus clear that one challenge is a lack of awareness of the time required for sample transport, so issues such as delays or misplaced samples are not proactively addressed. In addition, time is spent during this stage transcribing various information from the test order form, so information is being duplicated. This phase concludes with the preparation phase, in which the laboratory technician uses the test order information to prepare the sample for analysis. Informational gaps may cause delays at this point in the process because, just as the sample transport does not have a systematic workflow, the reporting of sample and test order errors back to the wards is similarly unstructured.

The analytical phase includes a single step, namely, the analysis of the laboratory sample. Information is generated as part of this process and is combined with information supplied during the pre-analytical phase (patient age, gender, etc.) to generate a result. The manual nature of matching the test results with the corresponding patient based on patient name is another informational step that can cause delays and potentially lead to reporting errors. The process of reporting the result back to the physician is the start of the post-analytical phase and, again, is not a formalised process. It could be performed by a variety of personnel at unspecified frequencies. Results are left in the laboratory entryway in cubby-style pigeon holes for pick-up. Often, when someone is sent from a ward to drop off laboratory samples, they will also pick up and deliver any results that are available for that ward. This means that critical results may not be reported to the wards in an expedited manner.

Table 3 presents the duration of the reporting stage for laboratory results from a 24-hour observation of the laboratory. Results were processed for all 25 patients who had test orders sent to the laboratory during the observation period. Test orders were reduced as the haematology instrument was not operational at that time and polymerase chain reaction results for outpatients were delivered through a different mechanism. Additionally, the hospital census was unusually low that day. Of the 25 results, 18 were collected during the 24-hour observation window, whilst seven remained in the pigeon holes. The average duration of the reporting stage was just under six hours, with significant variation. Two of the results were collected immediately because they were related to a critical patient and the laboratory called the ward when the results were available. On the other hand, over one-fifth of results spent more than 16 hours in the laboratory before being collected.

**TABLE 3:** Result reporting turnaround time from a 24-hour quality audit (*n* = 18).†

Variable	Time‡
Mean	5:51
Std. Err.	8:03
Range§	22:52

†, During the observation period, a total of 25 samples with test orders were sent to the laboratory. Of these, 18 results were collected and seven results remained uncollected at the end of the observation period.

‡, Times are given as hours; minutes. Time was calculated as the time that elapsed from when the laboratory results were made available in the laboratory until those results were collected by the originating clinical ward.

§, Difference between shortest and longest times.

## Discussion

The results present a multi-faceted depiction of laboratory testing in a hospital in a low-resource setting, focusing on both the demand for laboratory testing and the informational challenges in meeting that demand. With more than 240 000 tests conducted at the KCH laboratory during a one-year period, it is clear that laboratory services are very much in demand within the hospital, matching the rhetoric around the importance of accessible laboratory services in low-resource settings. However, the diagnostic process is information-intensive; and the quality audit and workflow analysis suggest that the capture, management, and transfer of this information are a significant barrier to maximising the laboratory's role at KCH.

The quality audit identified informational barriers in the collection and identification of samples. Almost one-sixth of samples were untestable because of incomplete or incorrect paperwork. The quality audit was conducted as a quality improvement effort and, as motivation for the study, laboratory employees articulated several challenges associated with incorrect or incomplete test orders. Firstly, patient details, such as age and gender, affect the interpretation of the results; and test orders that omit these data increase the likelihood of an interpretation error. Secondly, the time and date of the sample is particularly important for tests sent to either the microbiology or the biochemistry departments, so the absence of this information compromises the accuracy of the test. Finally, the current system for results reporting relies on ward information from the test order, so when this information is not included it is common for results to never reach the patient.

These pre-analytical errors and delays are similar to those found in other low-resource laboratory environments.^26^ Post-analytical delays were also evident from the analysis of results reporting, although the sample size was small and further examination is warranted. These inaccuracies have implications for both the hospital and the patients. Untestable samples equate to wasted resources, including the physical supplies for the sample, such as the syringe and collection vessels, as well as employee time involved in collecting the sample and communicating the error. For patients, these errors amount to delays in care; surgical patients may face delays in scheduled surgeries if laboratory results are not ready, whilst for others it may mean an extra night in the hospital. Efficient laboratory testing is particularly important for patients in critical condition, namely, those who face delays in life-saving care and/or empirical treatments prescribed in the absence of confirmatory laboratory results.

The findings of the quality audit also informed the interpretation of the data around the frequency of laboratory testing. The information about test volume reflected tests conducted by the KCH laboratory and therefore serves as a conservative measure of the demand for laboratory services at KCH. There are at least two reasons that demand for laboratory services may be greater than that shown in Table 1. First, samples deemed untestable may not always be corrected and re-sent to the laboratory, as the process for informing clinicians of untestable samples is unstructured and thus possibly lengthy; and clinicians may choose to pursue diagnosis and treatment without confirmatory laboratory results. Second, material shortages, such as reagents and properly functioning equipment, may prohibit the performance of certain kinds of tests.

The workflow analysis mapped the laboratory testing process at KCH to the standard stages of testing and identified the informational components of each stage. This exercise identified a lack of formalised workflow around the transport of samples from the wards to the laboratory and the reporting of results from the laboratory to the wards. The reporting delays were confirmed in the study of reporting times, although a limitation of this component of the results is the small sample size. These gaps suggest that one unique aspect of the informational challenges faced by laboratory systems is the geographic scale of the testing process. Unlike the paperwork issues identified in the quality audit, the workflow issue involves interactions amongst multiple agents across different departments.

The standard approach to laboratory testing does not necessarily reconcile these multiple players and environments. Information systems tend to have a departmental focus. For example, electronic medical records are patient-centric systems focusing primarily on managing clinical patient data. Whilst this often includes laboratory test results, electronic medical records functionality does not extend into the laboratory. Laboratory information systems, on the other hand, are typically specimen-centric systems, focusing on processes and workflows within the laboratory. In this scenario the generation of test orders and reporting of test results often falls within the gap between the electronic medical records and the laboratory information system.

We propose a system for supporting specimen management, increasing visibility into the status of all orders for both clinical as well as laboratory staff and following a model embraced by courier companies (DHL/Federal Express/UPS) to track and manage packages. This simple model has three main components: (1) the generation of a test order and associated paperwork; (2) the ability to monitor the status of the order, complete with exception alerts when applicable; and (3) electronic results reporting back to the ward. The approach of real-time monitoring of specimens as they move through each stage of the process is novel and is likely to have a higher impact in a low-resource setting, where challenges are arguably greater. In Table 4, we summarise problems identified in the KCH workflow and present proposed informatics interventions for each.

**TABLE 4:** Problems identified and proposed informatics solutions.

Problem identified	Proposed informatics intervention
Illegible labeling of specimens.	Computerised specimen labeling system to generate legible labels to be attached to specimens as they are obtained from patients on the wards.
Incomplete information on specimen label due to limited space on label to accommodate hand-written information (currently 16% of specimens discarded).	Computer generation of the label allows for smaller font and more compressed information.
Incomplete information on specimen label and/or test order form due to limited access to necessary information at the time of creating the order (currently 16% of specimens discarded).	Information more readily available when pulled directly from master patient index. Automatic date and time assigned by specimen labeling system.
Incomplete information on test order form (currently 16% of specimens discarded).	Duplicate label generated to be affixed to the test order form.
Inability to identify samples that are delayed in getting to the laboratory, or misplaced on the way to the laboratory.	Barcode scanning of samples arriving at the laboratory to timestamp their arrival. Alerts at the laboratory and nursing station when samples are ‘overdue’.
Duplication of order information recorded (once on the wards and again when specimen arrives in the laboratory).	Transmit the order electronically from the ward to the laboratory.
Difficulty tracking specimens by patient name (report from pharmacy staff).	Generation of accession numbers for each specimen, to be printed on specimen label and order form in both human-readable and barcode form.
No visibility for clinical staff when a sample has not passed viability inspection on arrival at the laboratory and needs to be withdrawn.	Status of all orders displayed on a Sample Status Dashboard at the relevant nursing stations. Alerts displayed for exceptions.
Delay in receiving the results due to batching of results and no person responsible for returning results to the wards (results currently wait ~6 hours on average before being returned to the ward).	Immediate delivery of results through electronic reporting to the nursing station (once released by the laboratory supervisor). Availability of results indicated on Sample Status Dashboard.
Delay in reporting critical results to the ward.	Critical results highlighted on Sample Status Dashboard at the nursing station.

Note: Informational barriers identified in the workflow analysis and potential informatics solutions.

The proposed informatics interventions address problems identified at KCH that would not generally be considered as being within the scope of a traditional laboratory information system implementation.^27^ This application of informatics to the pre-analytical and post-analytical phases is novel and these stages are natural targets for informatics interventions because they are information-intensive and often where errors arise.^17^ Such an approach, which recognises that much of the total testing process takes place outside the laboratory, may also improve the perception of laboratory testing amongst clinicians. Whilst clinicians in many of these settings have a tendency to circumvent laboratory testing in favour of more superficial, less accurate diagnostic signals, they may be more likely to opt for laboratory testing if they perceive it to be quick and accurate.^7,8^

We recognise that informatics interventions cannot solve all problems. Whilst our proposed informatics intervention does not prevent insufficient samples from being sent to the laboratory, it provides a formal mechanism for reporting and correcting those errors, potentially saving time in the laboratory testing process and improving the timely delivery of care. A variation of the proposed intervention could include decision support tools that remind the nurse preparing the order of the sample requirements (sample amount, container type, etc.).

The need for investment in laboratory infrastructure for disease prevention and control is recognised in the literature.^28^ Resource shortages, such as laboratory technicians, microscopes and access to electricity, are commonly cited as limiting factors in improving laboratory services.^29^ The informational challenges of laboratory testing in low-resource settings, whilst more challenging to identify than the resource limitations, will limit the impact of additional resources if unaddressed. This article synthesised an array of data about laboratory operations in a low-resource hospital setting, calling attention to these informational challenges. Next steps will include a larger-scale effort to document the pre-analytical and post-analytical phases at KCH and other low-resource hospital laboratories. In addition, whilst the proposed interventions are hypothetical at this point, we are in the early stages of modeling such systems.

### Limitations of the study

This study focuses on the informational challenges associated with laboratory testing at KCH and does not consider other challenges that may serve as limiting factors, such as the availability of reagents and other physical resources required for testing. Resolving informational challenges in the laboratory workflow may have a limited impact on overall laboratory performance if resource-based constraints are present. Furthermore, the findings from the results reporting study must be interpreted with caution, as the sample size was small.

### Recommendations

Future research will attempt to further quantify the workflows and challenges within the laboratory at KCH as well as those in other low-resource settings. In addition, evaluation of informatics solutions targeting the laboratory will speak to the extent that informational challenges are limiting the stature and role of the laboratory at KCH.

### Conclusion

In this article, we present a multi-faceted depiction of the laboratory testing process and informational challenges in a low-resource setting. One criticism of laboratory process analyses, even in more advanced settings, is the focus on the analytical phase.^27^ We presented evidence that encompassed all three stages of testing and identified two specific informational challenges: (1) complete testing paperwork; and (2) efficient, timely communication between the wards and laboratory. Indeed, these issues were identified because of the wider focus on the pre- and post-analytical phases, which capture the multiple players and locations involved in the complete laboratory testing process. Informational barriers and inefficiencies arose at the transition points, the transfer of responsibility from one role or location to another during the testing process. Whilst information such as test orders and results should support workflow and decision making, in this case it appears that challenges in information management are undermining these processes.

The plight of laboratory services in low-resource settings is at once loudly decried and woefully under-investigated. Here we presented examples of informational barriers in the pre- and post-analytical phases of the total testing process in a hospital in a low-resource setting – challenges which, by their nature, are predisposed to be mitigated or potentially even eliminated by informatics interventions. Future work will attempt to design, implement, and evaluate informatics solutions to these and other barriers to more efficient and integral laboratory systems in low-resource settings.

## Trustworthiness

The results presented represent the actual findings of the analyses described in the research method and design section, without alteration.

### Reliability and validity

The quality audit and turnaround time study reflect standard methods of measuring laboratory performance; and test volume is a standard measure of laboratory workload.
